# Mapping the Immune Cell Microenvironment with Spatial Profiling in Muscle Tissue Injected with the Venom of *Daboia russelii*

**DOI:** 10.3390/toxins15030208

**Published:** 2023-03-10

**Authors:** Ana K. de Oliveira, Patcharin Pramoonjago, Alexandra Rucavado, Christopher Moskaluk, Dilza T. Silva, Teresa Escalante, José María Gutiérrez, Jay W. Fox

**Affiliations:** 1 School of Medicine, University of Virginia, Charlottesville, VA 22908, USA; 2 Instituto Clodomiro Picado, Facultad de Microbiología, Universidad de Costa Rica, San José 11501, Costa Rica

**Keywords:** *Daboia russelii* venom, myonecrosis, desmin, inflammatory infiltrate, GeoMx^®^ Digital Spacial Profiler

## Abstract

Pathological and inflammatory events in muscle after the injection of snake venoms vary in different regions of the affected tissue and at different time intervals. In order to study such heterogeneity in the immune cell microenvironment, a murine model of muscle necrosis based on the injection of the venom of *Daboia russelii* was used. Histological and immunohistochemical methods were utilized to identify areas in muscle tissue with a different extent of muscle cell damage, based on the presence of hypercontracted muscle cells, a landmark of necrosis, and on the immunostaining for desmin. A gradient of inflammatory cells (neutrophils and macrophages) was observed from heavily necrotic areas to less damaged and non-necrotic areas. GeoMx^®^ Digital Spatial Profiler (NanoString, Seattle, WA, USA) was used for assessing the presence of markers of various immune cells by comparing high-desmin (nondamaged) and low-desmin (damaged) regions of muscle. Markers of monocytes, macrophages, M2 macrophages, dendritic cells, neutrophils, leukocyte adhesion and migration markers, and hematopoietic precursor cells showed higher levels in low-desmin regions, especially in samples collected 24 hr after venom injection, whereas several markers of lymphocytes did not. Moreover, apoptosis (BAD) and extracellular matrix (fibronectin) markers were also increased in low-desmin regions. Our findings reveal a hitherto-unknown picture of immune cell microheterogeneity in venom-injected muscle which greatly depends on the extent of muscle cell damage and the time lapse after venom injection.

## 1. Introduction

Snakebite envenoming is a neglected tropical disease that causes a significant number of deaths and disabilities on a global basis, particularly in impoverished rural settings of sub-Saharan Africa, Asia, and Latin America [1]. Owing to the great complexity and inter- and intraspecies variation in venom composition, there is a wide spectrum of clinical manifestations and complications associated with these envenomings [2,3]. Venoms of species of the family Viperidae, and of some of the family Elapidae, inflict severe tissue damage owing to the action of cytotoxic components and enzymes that degrade the extracellular matrix, mainly phospholipases A2 (PLA2s), zinc-dependent metalloproteinases (SVMPs), and cytotoxins of the three-finger toxin family [4,5,6].

Concomitantly with the direct toxic effects induced by these toxins in tissues, particularly in skin and skeletal muscle, venoms and toxins elicit a complex inflammatory response associated with an increase in vascular permeability leading to edema, the activation of resident cells in tissues, the recruitment and activation of inflammatory cells, and the generation of a plethora of inflammatory mediators that exert multiple actions on leucocytes and resident cells (see [7] for a review). In addition, damage-associated molecular patterns (DAMPs) released from damaged cells and degraded extracellular matrix components contribute to this inflammatory scenario by stimulating Toll-like receptors in innate immune cells [8,9,10].

Numerous investigations have analyzed the dynamics of inflammatory events occurring in vivo in rodent models after injection of venoms or purified tissue-damaging toxins. Most studies have used peritoneal lavage fluid after intraperitoneal injection [11] or of muscle tissue homogenates and exudates after intramuscular injection for subsequent analysis [12,13]. Other studies have addressed the changes in cytokines in blood after venom injections [14,15]. The processes of skin re-epithelization and skeletal muscle repair and regeneration following acute venom-induced tissue damage have also been investigated [16,17,18]. In turn, other studies have assessed the activation induced by venoms and toxins on a variety of cell types in vitro, such as macrophages [19,20,21], mast cells [22], fibroblasts [23], adipocytes [24], and synoviocytes [25]. This large body of information has led to the understanding that a complex interplay between cells and mediators is involved in tissue damage, inflammation, repair, and regeneration as a consequence of the actions of venoms and toxins.

In general, the methodologies followed in these studies have allowed for the quantification of inflammatory cells and mediators in samples collected from blood or tissue exudates and homogenates. However, there remains limited information on whether there is heterogeneity of these responses in different, specific areas of the affected tissues. It is likely that, depending on the venom/toxin concentrations in various regions of a particular tissue, the pathologic, inflammatory, and reparative events and mediators might vary. Thus, this introduces another level of complexity in terms of the spatial orientation of tissue responses to venoms that needs to be addressed. Furthermore, such tissue-heterogeneous scenario varies with time, with different time-courses of degenerative, reparative, and regenerative events, thus adding an additional element of complexity. It is, therefore, necessary to introduce novel methodologies that allow the analysis of such tissue heterogeneity in the tissue responses to the action of venoms [26,27].

To this end, we used Geomix^®^ Digital Spatial Profiler [28], in combination with histology and immunohistochemistry, to assess the host immune responses in the wound microenvironment of mouse skeletal muscle to the action of the venom of the Asian viperid snake *Daboia russelii*. These experimental approaches allow the spatial–temporal resolution of events in tissues and can be applied to and confirmed with paraffin-embedded samples, thus allowing the quantification of molecular markers vis à vis the analysis of the histological events in the tissues. Our findings reveal a hitherto-unknown pattern of spatial heterogeneity in tissue damage and inflammatory cells and mediators in muscle tissue, which largely depends on the extent of venom-induced damage and the different time points following tissue responses to venom.

## 2. Results

### 2.1. The Number of Inflammatory Cells Varies Depending on the Severity of Tissue Damage

**Figure 1:**
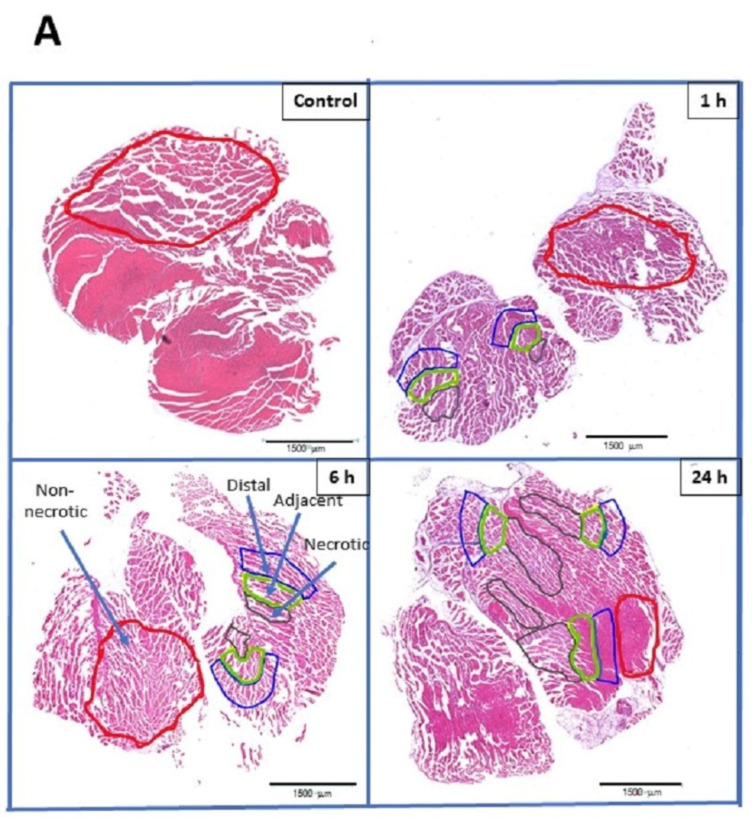

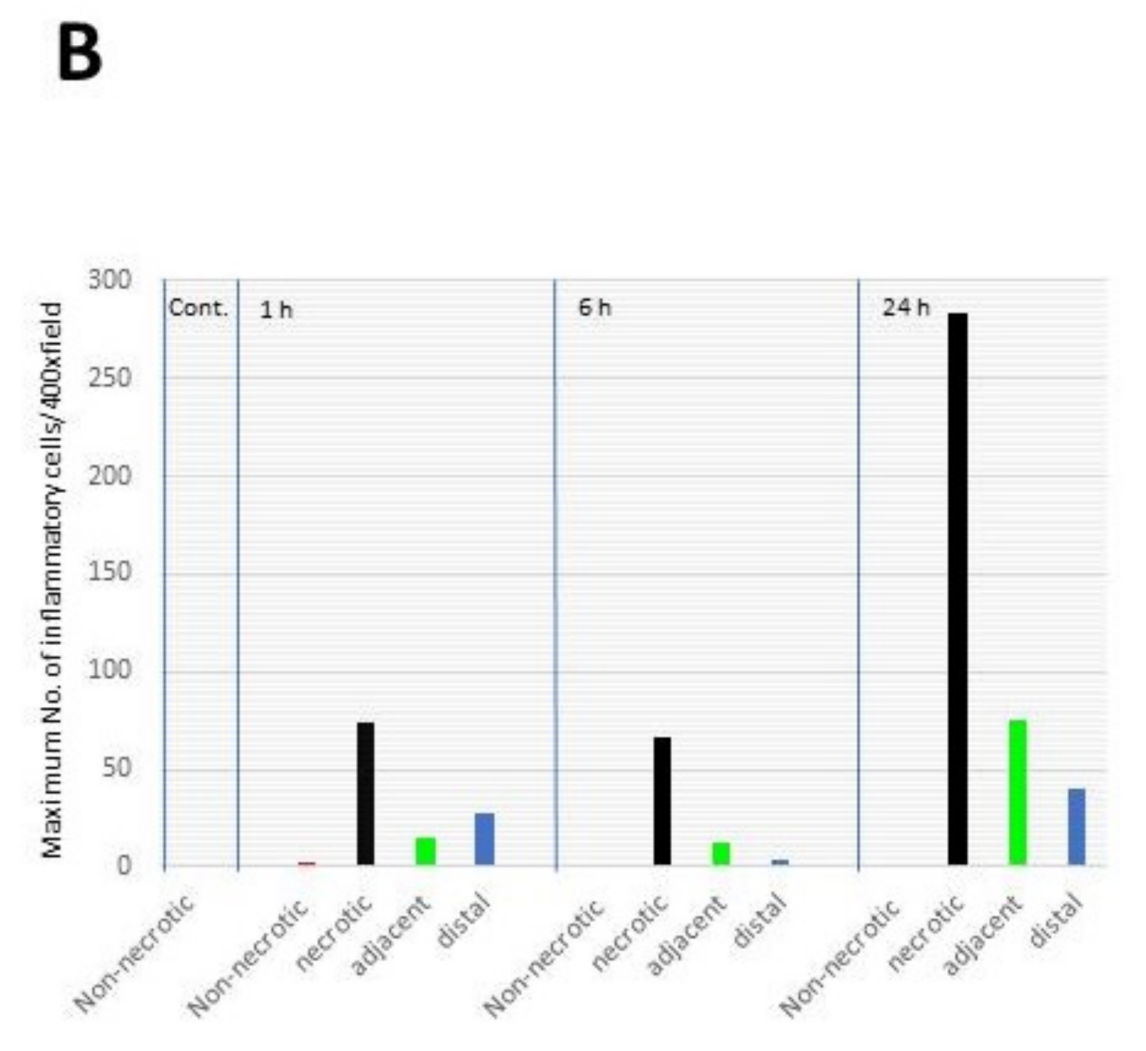
(**A**) Light micrographs of gastrocnemius muscle of mice injected with PBS (control) or with 30 µg of *D. russelii* venom, and sacrificed at either 1 h, 6 h, or 24 h after venom injection. Tissue was processed routinely and embedded in paraffin. Sections were stained with hematoxylin and eosin. Non-necrotic regions of the tissue are demarcated in red, whereas necrotic regions are demarcated in black. Regions adjacent and distal to the necrotic areas are demarcated in green and blue, respectively. Scale bar: 1500 µm. (**B**) Quantification of inflammatory cells in different regions of muscle injected with either PBS (control, non-necrotic) or with *D. russelii* venom. Tissue samples of envenomed mice were collected at 1 h, 6 h and 24 h and leukocytes were counted in non-necrotic, necrotic, adjacent, and distal regions. The quantity of inflammatory cells is expressed as the maximum number of inflammatory cells in each area per 400× microscopic field.

Skeletal muscle injected with *D. russelii* venom showed a heterogeneous pattern of tissue damage, i.e., myonecrosis, which was observed as early as 1 h after venom injection and increased at 6 h and 24 h. As previously described [12], there were areas with necrotic muscle cells, characterized by hypercontraction of myofibrils, and areas devoid of necrosis. In turn, areas of necrosis had various extents of damage as judged by the number of necrotic muscle cells and undamaged muscle cells. In order to assess whether the number of inflammatory cells, i.e., leukocytes and macrophages, differed depending on whether muscle cells were necrotic or not, four different areas were localized in the sections. These areas corresponded to (a) necrotic areas, characterized by the presence of abundant muscle cells displaying hypercontraction; (b) adjacent areas, corresponding to regions with few hypercontracted cells and with muscle cells not showing hypercontraction, but located in the vicinity of necrotic areas; (c) distal areas, corresponding to muscle cells not showing hypercontraction, but located close to the adjacent areas; and (d) non-necrotic areas, corresponding to regions away from the necrotic areas and characterized by normal muscle cell morphology (Figure 1A).

To this end, hematoxylin-and-eosin-stained sections were examined for the presence of inflammatory cells in these four different regions of the tissue. No inflammatory cells were observed in tissues from control mice. In contrast, muscle tissue from envenomed mice showed an increase in the number of inflammatory cells, which depended on the extent of damage in the areas being observed and on the time lapse when samples were collected. Necrotic areas with abundant hypercontracted muscle cells showed a higher number of inflammatory cells, as compared to adjacent, distal, and non-necrotic areas at 1 h and 3 h. At 24 h, the number of inflammatory cells was even more markedly increased. Inflammatory cells were also observed in adjacent and distal areas, with them being more abundant in the former when 24 h samples were analyzed (Figure 1B). Thus, there seemed to be a gradient of inflammatory cells from the necrotic areas to more distal areas in the affected tissue. In contrast, non-necrotic areas did not show inflammatory cells, similarly to control, non-envenomed muscle.

**Figure 2:**
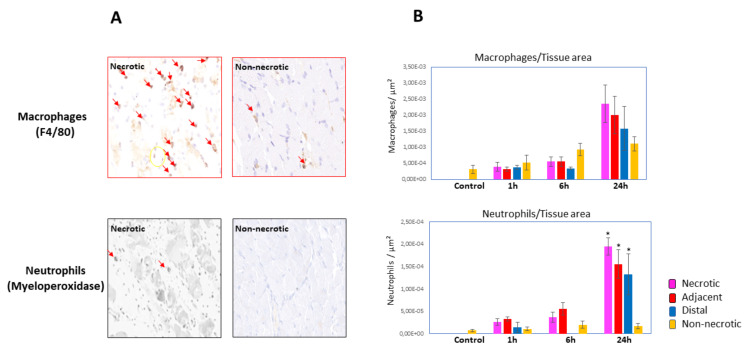
(**A**) Immunohistochemistry staining of macrophages (F4/80) and neutrophils (myeloperoxidase) in sections of muscle tissue injected with *D. russelii* venom. Notice the higher abundance of neutrophils and macrophages (arrows) in necrotic areas, as compared to non-necrotic ones. (**B**) Quantification of macrophages and neutrophils in control muscle injected with PBS and in the four regions of envenomed muscle (necrotic, adjacent, distal, and non-necrotic; see text for details). * *p* < 0.05 when compared to non-necrotic regions.

In order to expand these observations, sections were immunostained with antibodies specific for neutrophils (antimyeloperoxidase) and macrophages (anti-F4/80) (Figure 2A). No significant increase in the numbers of these cells were observed at 1 and 6 h, as compared to the control, nonenvenomed muscle. However, a significant increase in these inflammatory cells was observed at 24 h in the three areas corresponding to necrotic, adjacent, and distal regions, as described above (Figure 2A). A gradient in the number of neutrophils and macrophages was observed, going from necrotic to non-necrotic areas (Figure 2B,C).

### 2.2. GeoMx^®^ DSP Allowed the Quantification of Markers of Different Inflammatory Cells

**Figure 3:**
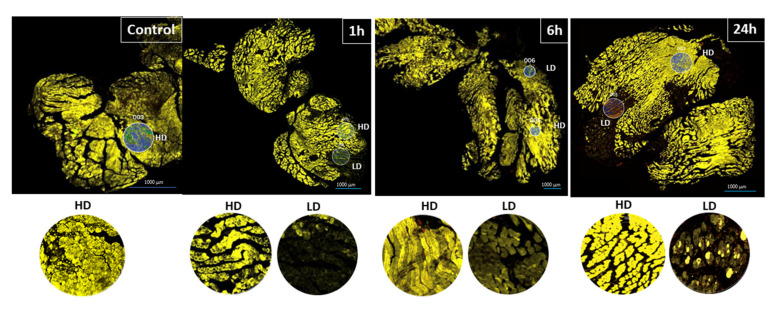
Sections of gastrocnemius muscles from mice injected with either PBS (control) or *D. russelii* venom. Samples from envenomed muscles were obtained 1 h, 6 h and 24 h after venom injection. After processing and embedding in paraffin, sections were obtained and stained with anti-desmin (yellow) and anti-CD-45 (red) antibodies, and with Syto 83 stain (for DNA, green). Depending on the intensity of the desmin staining, regions in the tissue were considered as either high-desmin (HD) or low-desmin (LD) staining, corresponding to non-necrotic regions and to damaged (necrotic) regions. Regions of interest (ROIs) were selected according to the density of desmin staining. ROIs chosen in this study ranged from 416 to 652 µm in diameter. Examples of ROIs are shown in the insets. Scale bar: 1000 µm.

In order to further understand the complexity of the inflammatory milieu in muscle tissue injected with *D. russelii* venom at various time intervals, sections were incubated with three fluorescent-labeled antibodies to detect desmin, leukocytes (CD45), together with nuclei stain (Syto 83), along with a cocktail of 48 oligonucleotide-labeled antibodies against different markers of inflammatory cells (Supplementary Table S1). To assess differences in the inflammatory cell markers depending to the extent of muscle tissue damage, regions of interest (ROIs) were selected according to the intensity of desmin staining (high and low), corresponding to areas of nonaffected muscle and damaged muscle, respectively (Figure 3).

**Figure 4:**
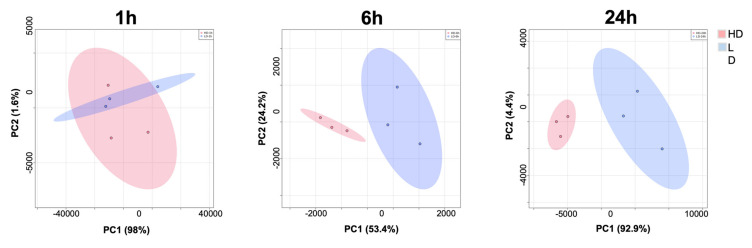
Principal component analysis (PCA) comparing high-desmin (HD) and low-desmin (LD) ROI in sections of muscle tissue of mice injected with the venom of *D. russelii*. Tissue samples were collected 1 h, 6 h, and 24 h after venom injection. The PCA analysis was performed using the prcomp package, and the scores plot between the selected PCs. The calculation is based on singular value decomposition. The data were generated using R version 4.2.2 (accessed on 31 October 2022 at https://www.metaboanalyst.ca).

**Figure 5:**
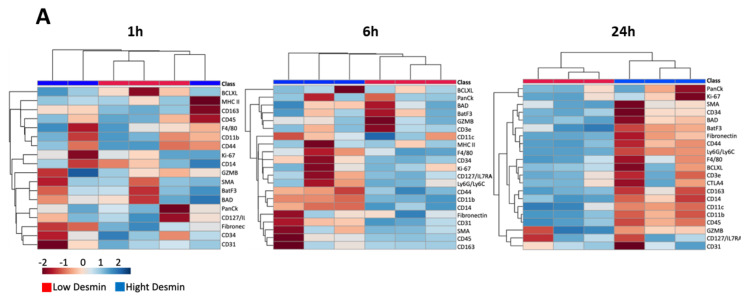

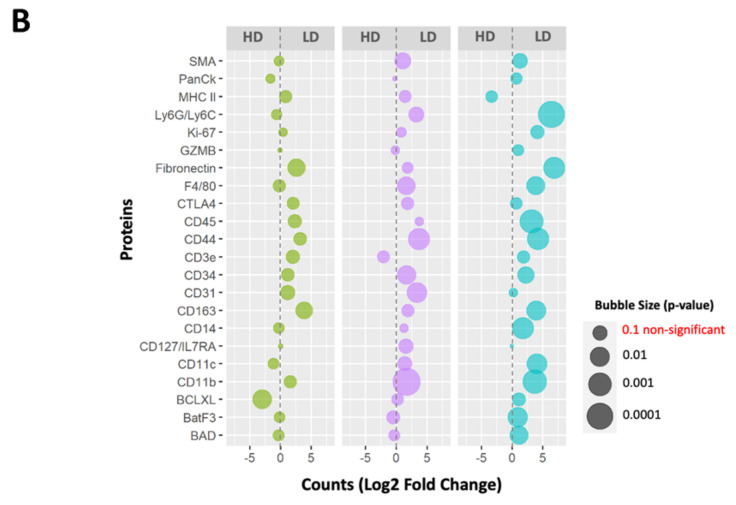
(**A**) Heat map analysis of the changes in cell markers in tissue samples from mice injected with the venom of *D. russelii* and collected 1 h, 6 h, and 24 h after injection. Missing values were replaced by data imputation (1/5 of the minimum positive value of each variable). Euclidean distance and single linkage methods were applied. Hierarchical clustering is performed with the hclust function in package stat using R version 4.2.2 (accessed on 31 October 2022 at https://www.metaboanalyst.ca). (**B**) Bubble Chart Analysis. The bubble chart depicts protein marker counts over time between low-desmin (LD) and high-desmin (HD) regions of the tissue. The color of each dot denotes the time interval, and the size of each dot denotes the *p* values comparing low-desmin and high-desmin regions (*p* < 0.05 denotes significance). The data were generated using R version 4.2.2 (accessed on 2 November 2022). The input data for this analysis is in Supplementary Table S4).

**Figure 6:**
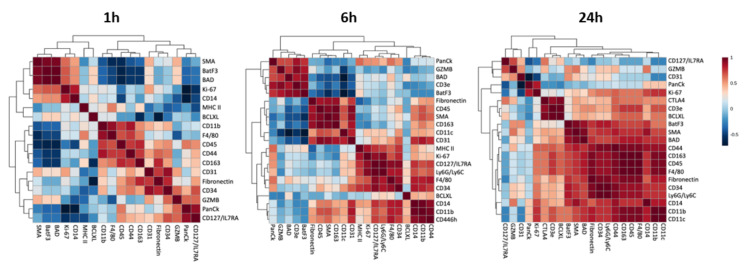
Correlations of the various cellular and tissue markers in muscle tissue injected with *D. russelii* venom. The proteins associations were analyzed over time between low-desmin and high-desmin regions. Red indicates a positive correlation and blue indicates a negative correlation. The input data for the analysis and the *p*-value from protein correlation are described in Supplementary Table S3. Proteins are clustered based on Spearman’s correlations using R version 4.2.2 (accessed on 31 October 2022 at https://www.metaboanalyst.ca).

A principal component analysis (PCA) revealed a difference between 1 h samples and 6 h and 24 h samples. In the former, there was an overlap between high-desmin and low-desmin areas, whereas in the latter, the data clustered separately (Figure 4). These findings agree with the heat map analysis in which no evident clustering was observed at 1 h but was clearly expressed in samples collected at 6 h and 24 h (Figure 5A). The bubble chart analysis revealed a significant increment in the majority of cellular markers in the low-desmin regions, with a gradient of increase along time, i.e., the highest changes in the 24 h samples (Figure 5B). The correlation analysis of the different cellular markers was carried out at the three time intervals. The correlation between markers increased as time passed, being highest in samples collected at 24 h (Figure 6).

**Figure 7:**
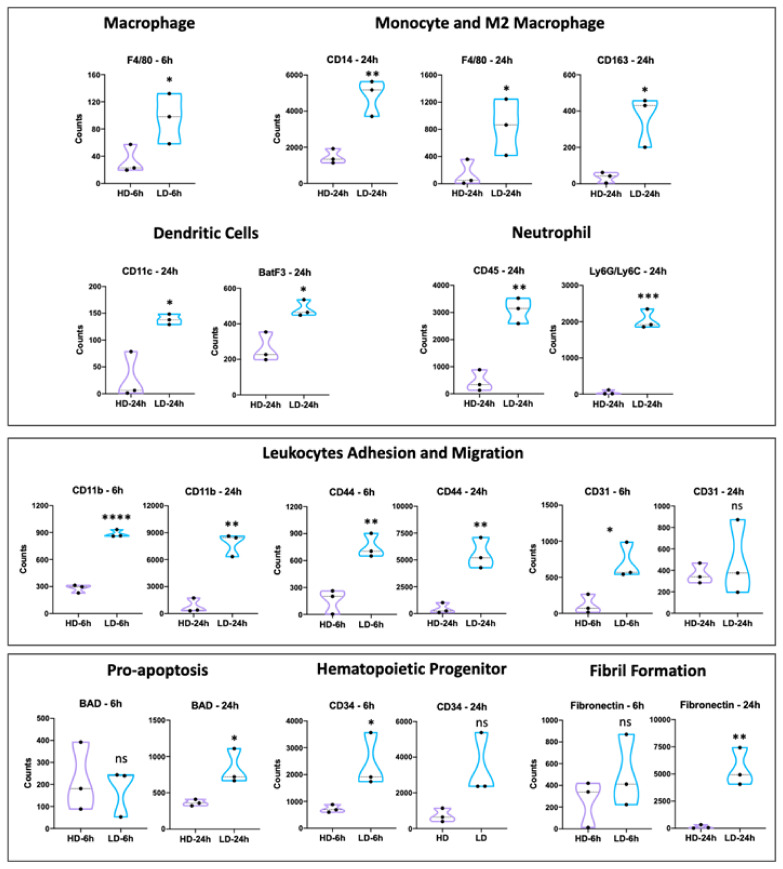
Comparison of different immune cell types, as judged by the expression of characteristic markers of each cell type, in low-desmin (LD) and high-desmin (HD) regions of the muscle tissue injected with *D. russelii* venom. Protein expression was assessed by protein count using GeoMx^®^ High-Plex Spatial Proteomics panel. Protein markers used were Neutrophils (6LysC-6LysG), dendritic cells (CD11c and BatF3), monocytes (CD14), and M2 Macrophages (F4/80 and CD163). In addition, proteins related to biological processes of regeneration (FN and CD34), apoptosis (BAD), adhesion, and migration of monocytes, macrophages, neutrophils, and T cells (CD44 and CD11b) were also identified. Groups were compared by unpaired *t*-test analysis using GraphPad Prism version 8.0.0 for MAC, (GraphPad Software, San Diego, CA, USA, www.graphpad.com, accessed on 22 January 2023). * *p* < 0.05, ** *p* < 0.01, *** *p* < 0.001, **** *p* < 0.0001.

Since the markers used are associated with different types of immune cells, we then compared different immune cell types in high- and low-desmin areas. In general, a clear trend was observed with significantly higher signals for markers of various cell types in low-desmin regions, i.e., in regions of muscle damage, particularly in samples collected 24 h after envenoming. These include markers of monocytes, macrophages, M2 macrophages, dendritic cells, neutrophils, leukocyte adhesion and migration markers, and hematopoietic precursor cells (Figure 7). We also assessed markers of apoptosis (BAD) and extracellular matrix (fibronectin), which were increased in 24 h samples, as compared to controls (Figure 7). In contrast, the following markers were not increase in low-desmin as compared to high-desmin regions: SMA, PanCk, MHCII, Ki67, GZMB, CTLA4, CD3e, and CD127/IL 7RA (Figure 7).

## 3. Discussion

The methods used in this study allowed for the analysis of the immune cell microenvironment in muscle tissue injected with a myotoxic snake venom. In particular, the GeoMx^®^ DSP platform provided a detailed assessment of markers of a variety of immune cells in various tissue environments which differed in the extent of muscle cell damage. In viperid snakebite envenomings, venom is often injected intramuscularly owing to the large size of fangs, which was our approach in this study. Once in the tissue, venom components are distributed and reach variable concentrations in different regions, thus affecting muscle cells at different extents depending on the concentration of myotoxic components, such as PLA2s, which directly damage the integrity of muscle plasma membrane [5]. As observed in this study, there are regions where the majority of muscle cells are necrotic, as evidenced by hypercontraction. These ‘ground zero’ areas are surrounded by regions where the number of necrotic muscle cells is reduced, while other more peripheral areas are devoid of damage. This microenvironment varies with time, as venom components further diffuse in the tissue and other alterations occur, such as microvessel damage leading to ischemia, also compromising the viability of muscle fibers [29]. This corroborates previous work which showed that, depending on the toxin concentration, a single cell type presents different responses, including necrosis, apoptosis, and cellular proliferation [30].

In parallel, a pronounced inflammatory reaction develops in the tissue affected by venom, characterized by increase in vascular permeability and an inflammatory infiltrate [7]. As shown in this study, the density and types of inflammatory cells also vary in regions having different extent of muscle damage. Inflammatory cells increased in necrotic muscle regions at 1 h and 6 h, while a higher increment was observed at 24 h. It was of interest to differentiate between neutrophils and macrophages at various times, since previous studies have demonstrated that neutrophils predominate early on and macrophages later on in the course of venom and myotoxin-induced muscle necrosis [31,32]. When using specific markers for these cell types it was observed that highest numbers of these cells were observed at 24 h. Interestingly, there is a gradient in the density of inflammatory cells from the core necrotic areas to adjacent and distal areas. However, even in distal areas having few necrotic fibers the numbers of neutrophils were higher than in non-necrotic areas of the tissue, i.e., where no hypercontracted muscle cells were observed. Thus, there was a correlation between the extent of muscle cell damage and the gradient of inflammatory cells in the tissue. Likewise, there was a time-dependent increase in the cellular infiltrate into affected muscle, reaching higher values at 24 h.

GeoMx^®^ DSP allowed an in-depth exploration of the composition of the immune cell infiltrate in damaged muscle, comparing highly affected regions (low desmin) and non-affected regions or areas affected to a lesser extent (high desmin). Markers of monocytes, macrophages, M2 macrophages, dendritic cells, neutrophils, leukocyte adhesion and migration proteins, and hematopoietic precursor cells were higher in low-desmin regions than in high-desmin regions, especially at 24 h, when the infiltrate was more abundant. Inflammatory cell infiltration depends on a highly orchestrated sequence of events which start with the generation of chemotactic stimuli from damaged tissue and resident cells, such as macrophages, and the concomitant generation of inflammatory mediators. This is followed by a process of rolling, adhesion, crawling, and transmigration of leukocytes across the vascular wall through the action of adhesion molecules, i.e., selectins, integrins and proteins of the immunoglobulin superfamily [33,34]. In support of this, our data indicated that several markers of leukocyte adhesion and transmigration were increased in low-desmin regions in our study.

Resident macrophages play a key role in the generation of chemotactic stimuli for the recruitment of neutrophils and monocytes in a model of toxin-induced muscle damage [32]. In the damaged tissue, leukocytes and macrophages become activated and synthesize reactive oxygen species and a plethora of inflammatory mediators. Activation occurs through the action of tissue components, i.e., damage-associated molecular patterns (DAMPs), inflammatory mediators released in the tissue by resident cells, and the direct action of venom toxins, i.e., venom-associated molecular patterns (VAMPs). Venoms and toxins are known to activate macrophages in vitro. In addition to other functions, leukocytes and macrophages play a key role in the removal of necrotic debris, which is critical for reparative and regenerative events that ensue after damage [35].

The phenotype of macrophages in necrotic muscle varies along time, with proinflammatory M1 macrophage predominating in the first wave of infiltration, and M2 pro-regenerative macrophages being predominant later on in the process [36]. Such variation in the macrophage landscape is required for successful muscle regeneration. This shift in the type of macrophage was described in a model of necrosis and regeneration induced by a myotoxic PLA2 homologue from the venom of *Bothrops jararacussu* [18]. We observed an increase in the marker CD-163, characteristic of M2 macrophages, by 24 h, in low-desmin regions, suggesting that the shift in macrophage subtype is already underway at this time interval. CD34, a marker of hematopoietic progenitor cells was elevated at 6 h and 24 h in low-desmin region, underscoring a process of inflammatory cell production.

Two markers of dendritic cells (CD11c and BatF3) were increased in low-desmin regions. Previous work described an increase in dendritic cells after 7 days in muscle damaged by notexin, a venom PLA2 [32]. In our study dendritic cells increased in low-desmin regions of muscle at 24 h after injury. The role of dendritic cells in this scenario remains unknown, although it suggests a connection between innate and adaptive immunity. Crotoxin, a venom PLA2, is known to modulate the function of dendritic cells [37] Dendritic cells have been associated with several autoimmune inflammatory myopathies [38,39]. When incubated with myoblasts in cell culture, dendritic cells are able to interact with them, inducing myoblast proliferation and migration, but inhibiting myotube differentiation [40]. Our observations stress the need to assess the role of dendritic cells in the overall processes of inflammation and muscle regeneration after venom-induced myonecrosis. In contrast to neutrophils, macrophages and dendritic cells, no significant differences between low-desmin and high-desmin regions were observed in several markers associated with T lymphocytes, i.e., GZMB, CTLA4, CD3e, and CD127, implying that these cells are not enriched in necrotic muscle.

A marker of apoptosis (BAD) was also investigated, being elevated in low-desmin regions by 24 h as compared to high-desmin regions. This raises the possibility that apoptosis is playing a role in this pathological model. Apoptosis has been associated with age-related skeletal muscle loss [41,42]. In addition, after infiltration in the necrotic muscle, neutrophils undergo apoptosis as part of their life cycle [32] and other cells in the inflammatory milieu may also undergo this type of cell death. Thus, the identity of cells undergoing apoptosis and the role of apoptosis in the overall muscle tissue damage in this model remain to be investigated. Changes in fibronectin were also assessed, being elevated in low-desmin regions by 24 h. Fibronectin is an extracellular matrix protein that plays roles in tissue repair and regeneration. In the case of skeletal muscle, the loss of this protein affects muscle regeneration in mice [43]. Thus, the higher expression of fibronectin in damaged areas may bear a relationship with the onset of muscle regeneration in our model.

In conclusion, our findings provide novel clues on the immune cell microenvironment in necrotic muscle tissue after the injection of *D. russelii* venom. In addition to neutrophils and macrophages, our data underscore the presence of dendritic cells in necrotic areas. A gradient of inflammatory cells was observed from heavily necrotic regions to outer regions having a lower extent of damage. These findings stress the need to consider the heterogeneity of tissue damage and inflammation when studying the effects of snake venoms in skeletal muscle and other tissues in order to have a more detailed and comprehensive view of these pathological events, as well as when considering tissue remodeling and regeneration.

## 4. Materials and Methods

### 4.1. Venom

*Daboia russelii* venom originating from specimens collected in Pakistan was purchased from LATOXAN (Code L1132A; Lot: 015.051; Portes.lès-Valence, France).

### 4.2. Experimental Model

Groups of mice of both sexes (CD-1 strain, 18–20 g) received an intramuscular injection, in the right gastrocnemius, of 30 µg of venom, dissolved in 50 µL of 0.12 M NaCl, 0.04 M phosphates, pH 7.2 (PBS). Control mice were injected with 50 µL of PBS only. At the time intervals of 1 h, 6 h, 24 h following injection, groups of three mice were sacrificed via CO_2_ inhalation, and a sample of the injected gastrocnemius muscle was excised and added to 10% formalin solution in water. After 48 h fixation, the routine processing of tissues was performed, followed by embedding in paraffin. The protocols of experiments using mice were approved by the Institutional Committee for the Care and Use of Laboratory Animals (CICUA) of the University of Costa Rica (approval number CICUA-032-2020) and meet the International Guiding Principles for Biomedical Research Involving Animals (CIOMS).

### 4.3. Histology and Immunohistochemistry

Muscle tissue sections (4 µm thick) were stained with hematoxylin and eosin for histological observation of muscle damage and quantification of inflammatory infiltrate. Necrotic muscle fibers were identified by the hypercontraction of myofilaments, and inflammatory infiltrate was assessed by counting the number of polymorphonuclear leucocytes, mostly neutrophils, and macrophages in the necrotic and non-necrotic regions. In order to assess in more detail the presence of inflammatory infiltrate in muscle tissue having various extents of damage, areas of the tissue were categorized as necrotic, adjacent, distal and non-necrotic (see Results section for a detailed explanation). Similar sections were used for immunohistochemistry analysis. Immunohistochemistry was performed on a robotic platform (Ventana discover Ultra Staining Module, Ventana Co., Tucson, AZ, USA). A heat-induced antigen retrieval protocol set for 64 min was carried out using a TRIS–ethylenediamine tetraacetic acid (EDTA)–boric acid pH 8.4 buffer (Cell Conditioner 1). Endogenous peroxidases were blocked with peroxidase inhibitor (CM1) for 8 min before incubating the tissues for 60 min at room temperature with prediluted myeloperoxidase antibody (Novus Biologicals, Cat# NBPI-82574), neutrophil marker, or F4/80 antibody (Bio-Rad, Cat # MCA-497), a macrophage marker, at 1:80 dilution. The antigen–antibody complex was then detected by DISCOVERY OmniMap anti-Rb and anti-Rat HRP RUO system, respectively, with DISCOVERY ChromoMap DAB Kit (Ventana Co.). All the slides were subsequently counterstained with hematoxylin; they were dehydrated, cleared, and mounted for the analysis. The immunohistochemically stained sections were next scanned with Hamamatsu NanoZoomer-S360 digital whole slide scanner, and the images were analyzed with Visiopharm software through the use of a custom-made algorithm for neutrophils and macrophages, on the basis of staining intensity and background staining.

### 4.4. GeoMx^®^ Digital Spatial Profiler

The GeoMx^®^ Digital Spatial Profiler (DSP) is a novel platform developed by NanoString [28]. This product relies on antibodies coupled to photocleavable oligonucleotide tags. After probes hybridize to targets in slide-mounted tissue sections, the oligonucleotide tags are released from discrete regions of the tissue via UV light exposure. Released tags are quantitated by nCounter technology. Counts are mapped back to tissue location, yielding a spatially resolved digital profile of analyte abundance.

In brief, slides from tissue sections obtained as described above were deparaffinized, subjected to antigen retrieval, and incubated overnight with three fluorescent-labeled visualization antibodies to detect muscle fibers (desmin), leukocytes (CD45), and nuclear stain (Syto 83), along with a cocktail of 48 oligonucleotide-labeled antibodies to detect a variety of cellular markers (Supplementary Table S1). Eighteen regions of interest (ROI) from 416 to 652 μm diameter were selected based on desmin staining intensity to generate low-desmin (LD) and high-desmin (HD) areas from skeletal muscle tissue of control mice injected with PBS and from mice injected with *D. russelii* venom harvested at 1 h, 6 h, and 24 h. Desmin was selected as a marker of viable muscle cells, since this intermediate filament protein is rapidly lost in cells affected by muscle-damaging venoms and toxins [44,45]. Thus, immunostaining of desmin allowed the identification of affected and non-affected regions in muscle tissue. After UV light illumination of the ROIs, the eluent was collected via microcapillary aspiration and transferred into individual wells of a microtiter plate. Once the 18 ROIs were processed, indexing oligos were hybridized to NanoString optical barcodes for digital counting on the nCounter. Digital counts from barcodes corresponding to protein probes were then normalized to housekeeping controls, S6 and GAPDH, of their defined ROIs and, subsequently, background was subtracted using the IgG controls (rabbit or mouse depending on the origin of the antibody used).

### 4.5. Data Analysis

The effect of *D. russelii* venom was analyzed over time by comparing the low-desmin (LD) to high-desmin (HD) regions. After data normalization using GeoMx^®^ DSP, the samples with a count less than or equal to the background value were excluded from the analyses. Missing values were replaced by data imputation of 1/5 of the minimum positive value of each variable (Supplementary Table S2).

The datasets were processed using MetaboAnalystR 3.0 platform or R package (v4.2.2). PCA was performed based on the normalized data using MetaboAnalystR 3.0. Spearman correlations between the LD (non-necrotic regions) and HD regions were calculated using the R package (v4.2.2). The confidence interval of the significant correlation was set to 0.95. Proteins with more than 3 missing values were excluded from this analysis. The correlation *p*-value is indicated in Supplementary Table S3.

Statistical analyses of the data generated herein were performed using R version (v4.2.2) and GraphPad Prism version 8.2.1 (GraphPad; https://www.graphpad.com, accessed on 22 January 2023), using a parametric unpaired Student’s *t*-test. Statistical significance was established at *p* ≤ 0.05.

## Data Availability

Data are submitted on 16 January 2023 at GSE222977, https://www.ncbi.nlm.nih.gov/geo/query/acc.cgi?acc=GSE222977 (accessed on 22 January 2023).

## Supplementary Materials

The following supporting information can be downloaded at: https://www.mdpi.com/article/10.3390/toxins15030208/s1, Table S1: GeoMx panel of markers used. Table S2: Normalization Data. Table S3: Protein correlation. Table S4: B chart input data.
